# SMILE: Semi-supervised multi-view classification based on dynamical fusion

**DOI:** 10.1371/journal.pone.0320831

**Published:** 2025-05-20

**Authors:** Hui Yang, Linyan Kang, Xun Che

**Affiliations:** 1 School of Cyberspace Security, Hunan College of Information, Changsha, Hunan, China; 2 School of Science, Hangzhou Dianzi University, Hangzhou, Zhejiang, China; 3 School of Computer Science and Engineering, Nanjing University of Science and Technology, Nanjing, Jiangsu, China; Bournemouth University, UNITED KINGDOM OF GREAT BRITAIN AND NORTHERN IRELAND

## Abstract

Semi-supervised multi-view classification plays a crucial role in understanding and utilizing existing multi-view data, especially in domains like medical diagnosis and autonomous driving. However, conventional semi-supervised multi-view classification methods often merely fuse features from multiple views without significantly improving classification performance. To address this issue, we propose a dynamic fusion approach for **S**emi-supervised **M**ult **I**-view c **L**assification (SMILE). This approach leverages a high-level semantic mapping module to extract discriminative features from each view, reducing redundancy features. Furthermore, it introduces a dynamic fusion module to assess the quality of different views of different samples dynamically, diminishing the negative impact of low-quality views. We compare our method with six competitive methods on four datasets, exhibiting distinct advantages on the classification task, which demonstrates significant performance improvements across various evaluation metrics. Visualization experiments demonstrate that our approach is able to learn classification-friendly representations.

## 1 Introduction

With the development of multimedia technology, most real-life data exists in the form of multi-view/multi-modality. For example, during autonomous driving, different sensors perceive the surrounding environment, such as ultrasonic radar, cameras, millimeter-wave radar, etc., and the data collected by each sensor is regarded as a view [1–5]. A video consists of audio, images, and text, with each medium acting as a view [6]. Fully understanding and utilizing these multi-view data can better mine data and drive innovation and progress. However, with the continuous increase in manual annotation costs and massive data, there is an urgent need for a more effective way to process multi-view data [7–10]. Therefore, this work focuses on semi-supervised multi-view classification.

The main challenge to semi-supervised multi-view classification is: how to fully utilize a small amount of labeled data and a large amount of unlabeled data to obtain a complete (containing cross-view shared information and complementary information) multi-view representation. Existing semi-supervised multi-view classification methods can be mainly divided into traditional methods and deep learning-based methods [11, 12]. Traditional methods mostly rely on the shared representation after multi-view fusion. Due to the limited ability of shallow methods to extract high-level semantic information from data, their classification performance depends highly on the original data. With the rapid development of deep learning, deep semi-supervised multi-view classification methods utilize the powerful representation ability of deep models to learn multi-view fusion representations that are beneficial for classification, thus overcoming the shortcomings of traditional methods and making deep learning-based semi-supervised multi-view classification attract tremendous attention in the community [13, 14].

Existing deep learning-based semi-supervised multi-view classification methods usually utilize autoencoders or convolutional neural networks to extract features from multiple views and maximize the shared representation to obtain fusion representations, and then use semi-supervised strategies based on the fused representations to generate supervised information for unlabeled data [15, 16]. Although these methods have made some progress, they simply concatenate the features of multiple views or obtain fusion features through a neural network, and cannot effectively estimate the contributions of different views. Multi-view data is composed of data from different sources, and it contains not only shared information but also a large amount of specific view information. The informativeness of each view in different samples is different. Therefore, dynamically fusing different views for each sample is beneficial to improve the quality of the fusion representations, thereby improving the classification performance of the model.

To address this issue, we propose a novel method called **S**emi-supervised **M**ult **I**-view c **L**assification based on dynamic fusion (SMILE). The method introduces a view-specific autoencoder (AE) for each view to extract low-level features. Since these low-level features are heavily tied to the reconstruction task and are not ideal for classification, we incorporate a high-level semantic mapping head for each view to transform these features into more suitable high-level features for classification. Additionally, a view confidence module evaluates the informativeness of each view for different samples, allowing for the dynamic fusion of views. This approach mitigates the negative impact of low-quality views, ensuring the model remains robust to variations in view quality. The main contributions of this work are summarized as follows:

- This work proposes a novel algorithm for semi-supervised multi-view classification that is robust to low-quality views and significantly improves classification performance.
- The introduced view confidence module dynamically evaluates the informativeness of each view of each sample, refines discriminative features and dynamically fuses features of multiple views.
- It is proved through visualization experiments that the proposed method can learn fusion representations with clear classification structure; ablation experiments prove the importance of the dynamic fusion module and the high-level feature mapping head; classification performance comparison experiments with 6 competitive methods on 4 benchmark datasets show the effectiveness and superiority of the proposed method.

## 2 Related work

Most data in real life exists in the form of multi-view/multimodal data. For example, a video contains three modalities: audio, image, and text; data collected by different sensors in autonomous driving constitute multiple modalities; multiple views in medical diagnosis are formed by different medical images (such as X-ray, MRI, and CT scans) of the same lesion [17–21]. Multi-view data contains richer information than single-view data and fully exploring multi-view data can better mine the knowledge behind the data, thereby it can promote social development and progress [22, 23]. However, with the explosive growth of data, existing data commonly consists of numerous unlabeled data and merely a small amount of labeled data. Due to the high cost of manual labeling, how to fully utilize the small amount of labeled data and the numerous unlabeled data has become a hot research topic and difficulty in the current research [24–27]. In the face of the dual challenges of multi-view and few labels, there is an urgent need to propose a more effective method, so semi-supervised multi-view learning has emerged and become a research focus.

Existing semi-supervised multi-view learning roughly category two groups one is traditional methods and the other is deep learning-based methods. Traditional methods primarily fall into the following categories: (1) co-training techniques [19, 28, 29]; (2) graph-based strategies [30–32]; and (3) regression-based approaches [33–35]. Co-training, initially developed for dual-view data, starts by training a classifier on labeled data. It then assigns labels to the unlabeled data in each view. Subsequently, the most confidently predicted samples from one classifier are added to the training set of the other classifier, and this cycle is repeated [36]. Graph-based methods use unlabeled and labeled data as vertices of the consensus graph, and then propagate the labeling information using edges. For example, these methods [31, 32] initially construct a graph for each view, then learn the weights of the views to obtain a consensus graph, and use label propagation to predict labels for unlabeled data. In line with this, Nie *et al*. [31] propose a parameter-free method to simultaneously learn the consensus graph matrix, the commonly labeled indication matrix, and the view weights. Regression-based learning techniques are employed to derive projection matrices for each view, thereby exploring view-specific complementary information. They utilize the labeled matrix as a cross-view regression target to investigate consistency among views [33]. While these methods demonstrate significant advancements, they are not without their limitations. Co-training approaches tend to overlook the inherent diversity of multiple views, treating them as equivalent. This oversight not only fails to rectify the inaccuracies produced by low-quality views but also exacerbates these errors within the model, ultimately resulting in a decline in performance. Furthermore, these methods are constrained in their ability to perform representation learning and are unable to effectively explore high-level semantic information within the data. In contrast, deep learning-based approaches excel outstanding in representation learning have garnered significant attention [6, 37, 38].

Pseudo-labeling methods demonstrate superior performance compared to many other techniques in depth-based semi-supervised learning due to their lightweight and effective approach. This advantage may be attributed to the substantial number of parameters that require tuning in deep neural networks; an increased number of parameters necessitates a greater volume of labeled data. Pseudo-labeling methods provide labeled data directly, which is particularly advantageous for deep learning models [25, 27]. For example, Wang *et al*. [1] proposed generating pseudo-labels on the fused representation of multiple views as supervised information to guide the learning of the single-view representation. With more supervised information, the learning of representations for individual views is thus improved thus facilitating the generation of better-fused representations. It follows that the quality of pseudo-labeling depends on the quality of the input representations. However, these methods typically employ straightforward fusion techniques to integrate features from multiple views, neglecting the variability in informativeness across different samples. This oversight diminishes the distinguishability of the fused representations. Consequently, the representations generated by existing methods fail to mitigate the adverse effects of low-quality views, resulting in a limited acquisition of discriminative features. This limitation ultimately constrains the enhancement of classification performance.

Unlike these approaches, this study dynamically evaluates the informativeness of different views for different samples and then dynamically fuses the features of multiple views to generate classification-friendly representations.

## 3 Our method

### 3.1 Prelimilary

**Fig 1:**
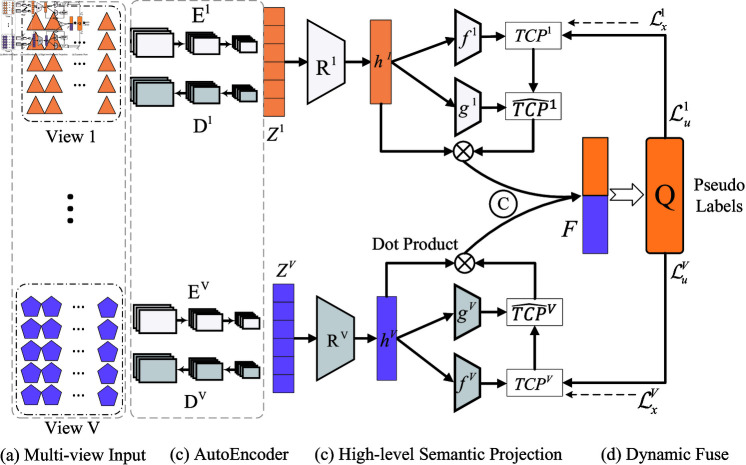
Illustrated of our proposed SMILE, which pipeline as follows: ( **a**) input multi-view data; ( **b**) obtain view-specific representations through view-specific autoencoders; ( **c**) high-level semantic projection module projects view-specific representations into high-level features; ( **d**) dynamic fuse multiple view-specific high-level features.

In this section, the proposed semi-supervised multi-view classification algorithm based on dynamic fusion is presented. For ease of exposition, this section begins with a description of the notation involved. Given a multi-view dataset 𝒟, where labeled data is defined as $𝒳=\{\{{𝐱}_{i}^{1},{𝐱}_{i}^{2},\ldots ,{𝐱}_{i}^{V}\},{𝐲}_{i}{\}}_{i=1}^{L}$, where *V* denotes the number of views, **y** is the label corresponding to the current sample, and *L* is the number of labeled data. Unlabeled data is defined as $𝒰=\{{𝐱}_{i}^{1},{𝐱}_{i}^{2},\ldots ,{𝐱}_{i}^{V}{\}}_{i=1}^{U}$, where *U* is the number of unlabeled data. ${𝐱}_{i}^{v}\in {\mathbb{R}}^{d_{m}}$ denotes the feature dimension of the *v*-view of the *i*-th sample is *d*_*m*_. The purpose of semi-supervised multi-view classification is to predict correct classification results for unlabeled samples using a small amount of labeled data and a large amount of unlabeled data. The framework of the proposed method in this work is shown in Fig 1.

### 3.2 Multi-view data reconstruction

The raw multiview data has a large number of redundant features, so representative features are first learned from the raw data. AutoEncoder is a model that maps raw data to feature space and is widely used because of its simplicity and effectiveness [39, 40]. Therefore, in this work, view-specific AutoEncoders are designed for each view to extract the features of each view. Specifically, for the *v*-view of sample *i*, E(·) is introduced as an encoder nonlinear function to map each view into view-specific representations:

$${𝐳}_{i}^{v}=E^{v}({𝐱}_{i}^{v}),$$ (1)

where ${𝐳}_{i}^{v}\in {\mathbb{R}}^{d_{v}}$ is the obtained low-level features that the decoder will reconstruct ${𝐳}_{i}^{v}$ to obtain the reconstructed view. Specifically, the decoder is denoted by $D^{v}(\cdot )$ and the obtained reconstructed view is denoted as:

$${\hat{x}}_{i}^{v}=D^{v}({𝐳}_{i}^{v}).$$ (2)

This work utilizes reconstruction loss ${ℒ}_{r}$ to optimize this process, which defined as:

$$L_{r}=\frac{1}{L+U}\sum_{i=1}^{L+U}\sum_{v=1}^{V}\|{𝐱}_{i}^{v}-{\hat{x}}_{i}^{v}{\|}_{2}^{2}.$$ (3)

### 3.3 Dynamic multi-view fusion

The low-level features of a single view extracted by the autoencoder, which contains most of the features relevant for reconstruction, will face some challenges if the classification is performed directly on the low-level feature space. To obtain more class-specific features to facilitate the performance improvement of classification, we add an additional fully connected layer on the low-level features to map them to the high-level representation space ${𝐡}_{i}^{v}=R^{v}({𝐳}_{i}^{v})$, ${𝐡}_{i}^{v}$ is the obtained high-level features, and $R^{v}(\cdot )$ is the high-level semantic projection module of the *v*-view.

In multi-view data, the informativeness of each view of different samples is invariant [41, 42], therefore, understanding the variation of the informativeness for different samples is the key to multi-view classification, which is related to the ability of the model to adapt to the quality of the modality changes. Inspired by the literature [43], this work introduces the True-Class-Probability (TCP) [44] to quantify the categorization confidence of different views, which is closely related to the amount of categorization information of the views. When the current view classification confidence is low, it indicates that the current classification is unreliable, which correspondingly means that the view contains less informative information. In order to obtain the classification confidence of the views, for each view *v*, this work designs a classifier $f^{v}$ as a probabilistic model that transforms the observed samples ${𝐱}_{i}^{v}$ into a predictive distribution $P^{v}(y|x^{v})=[p_{1}^{v},p_{2}^{v},\ldots ,p_{C}^{v}]$, *C* is the number of classes. The classifier can be trained using the maximum likelihood estimation framework to minimize the KL (Kullback-Leibler) divergence between the predicted distribution and the true distribution:

$${ℒ}^{cls}=-\sum_{v=1}^{V}\sum_{c=1}^{C}{𝐲}_{c}log(P_{c}^{v}),$$ (4)

where Eq. 4 is also known as the cross-entropy function. The maximum class probability can be extrapolated to ${\text{MCP}}^{v}=max\{p_{1}^{v},p_{2}^{v},\ldots ,p_{C}^{v}\}$, and can also be considered as, the classifier’s confidence in the current prediction. Although this is effective in classification, its tendency is to lead to overconfidence in the model (assigning higher confidence scores for error predictions as well). Therefore, in order to obtain more reliable classification confidence, TCP is used in this work. Unlike MCP which utilizes the maximum softmax output as a measure of confidence, TCP uses the Softmax output probability that corresponds to the true label as its confidence. Specifically, for each view *v*, the corresponding prediction distribution $p^{v}(𝐲|{𝐱}^{v})=[p_{1}^{v},p_{2}^{v},\ldots ,p_{C}^{v}]$ and label **y** is obtained, then the TCP can be formalized as:

$${\text{TCP}}^{v}=\sum_{c=1}^{C}{𝐲}_{c}p_{c}^{v},$$ (5)

where (·) denotes the inner product, and when the model predictions are correct, the output of TCP agrees with the output of MCP. At this point, both TCP and MCP are maximal Softmax outputs and both reflect classification confidence well. When misclassified, however, the TCP is a better reflection of classification because it is more likely to approach a lower value, reflecting the fact that the model tends to make incorrect predictions. Although TCP gives more reliable confidence, it cannot be used directly in the estimation of the test phase and unlabeled samples because of the need for real labeling information, so a confidence regression network $g^{v}:{𝐡}^{v}\to {\text{TCP}}^{v}$ is introduced for each view *v* to estimate TCP because TCP∈(0,1), therefore, a sigmoid activation function is added to the last layer of the network and the confidence regression network is trained with loss:

$${ℒ}^{conf}=\sum_{v=1}^{V}\|{\hat{\text{TCP}}}^{v},{\text{TCP}}^{v}{\|}_{2}^{2}+{ℒ}^{cls},$$ (6)

where ${\hat{TCP}}^{v}=g^{v}({𝐡}^{v})$, then the TCP can be approximated with a view-specific classifier and a confidence regression network. Thus the fusion representation of multiple views is shown below:

$$𝐅=[{\hat{TCP}}^{1}{𝐡}^{1},\ldots ,{\hat{TCP}}^{V}{𝐡}^{V}],$$ (7)

where [·,·] denotes a concatenation operator.

### 3.4 Objective function

In this work, for the fusion representation, an additional classifier is trained with cross-entropy loss to get the final classification result *p*, therefore, the supervised loss in this work is formulated as follows:

$${ℒ}_{x}=\frac{1}{L}\sum_{l=1}^{L}(-\sum_{c=1}^{C}{𝐲}_{c}log(p_{c})+{ℒ}^{conf})$$ (8)

For unlabeled samples, this work employs a threshold-based method to evaluate the reliability of predicted pseudo-labels and selects trustworthy pseudo-labels for training. The unsupervised loss can be defined as the cross-entropy loss between the pseudo-labels and the model predictions:

$${ℒ}_{u}=\frac{1}{U}\sum_{u=1}^{U}\sum_{v=1}^{V}1(max(q_{u})\leq \tau )H(q_{u},p^{v}(𝐲|{𝐱}_{u})),$$ (9)

where the pseudo-labels with a maximum class prediction probability equal to or exceeding the threshold τ are deemed credible, and τ is a predefined hyperparameter. H(·) denotes the cross-entropy loss. Thus the objective function of the proposed method in this work can be defined as:

$$ℒ={ℒ}_{x}+\lambda ({ℒ}_{u}+{ℒ}_{r}),$$ (10)

where λ is the balance factor to balance different losses, which is set to 1 in the experiments in this work.

## 4 Experimental setup

### 4.1 Datasets

- **Handwritten** (https://archive.ics.uci.edu/ml/datasets/Multiple+Features) is a handwritten digits dataset comprises 2,000 samples, categorized into 10 classes, and includes six different views.
- **Scene15** [45] is a dataset that consists of images categorized into 15 indoor and outdoor scene categories. In this work, we employ GIST, PJOG, and LBP features, utilizing these three views with a total of 4,485 samples to construct the dataset.
- **Out-Scene** [46] dataset is specifically designed for scene classification tasks and comprises 2,688 outdoor scene images, which are categorized into eight classes. Each sample within the dataset includes four views.
- **GRAZ02** (http://www.emt.tugraz.at/pinz/data/GRAZ_02) is a widely utilized benchmark for object categorization and recognition tasks, consisting of 1,474 samples across four classes, with each sample encompassing six views.

### 4.2 Comparison methods

This study undertakes comparative experiments to assess and compare the proposed method against six state-of-the-art semi-supervised multi-view classification methods.

- **AMGL** [31] is developed for multiview clustering and semi-supervised tasks, enabling the automatic learning of optimal graph weights without the need for additional parameters. This approach incorporates heterogeneous features to align with actual data distributions and ensures the attainment of a globally optimal solution.
- **MLAN** [32] concurrently executes clustering or semi-supervised classification alongside local structure learning. The model autonomously determines optimal weights for each view without the need for explicit weight specifications or penalty parameters. Furthermore, it is capable of producing reliable graphs even in the presence of noisy data.
- **MVAR** [33] employs regression-based loss functions that utilize the $\ell_{2,1}$ matrix norm, integrating them in a linear fashion. It features an efficient and convergent algorithm designed for the minimization of the non-smooth $\ell_{2,1}$-norm, rendering it appropriate for large-scale datasets. Furthermore, MVAR automatically adjusts weights to accommodate low-quality views and streamlines the prediction process for new data.
- **JCD** [30] learns both a common label matrix and view-specific classifiers. It proposes a novel probabilistic square hinge loss to handle uncertain sample contributions and uses power mean to weight losses from different views.
- **LACK** [47] presents a label-driven auto-weighted approach that assesses the significance of views through labeling rather than through data representation. This methodology enables LACK to acquire labels with enhanced accuracy in view weights by decomposing the overarching problem into three smaller, more manageable sub-problems that can be solved efficiently.
- **IMvGCN** [48] integrates Graph Convolutional Networks (GCN) with multi-view learning to enhance interpretability and performance. It combines reconstruction error and Laplacian embedding to address multi-view learning from both feature and topology perspectives.
- **SMILE-L** is our proposed method that the model just trained with unlabeled data.

#### 4.2.1 Experimental details

In the experiments, the view-specific feature extraction network is implemented as a 3-layer Multilayer Perceptron (MLP). We use the Adam optimizer with weight decay to adjust the learnable parameters, setting the learning rate to 1*e*–3. The balancing parameter λ is selected from {1,0.5,0.1}, and the threshold τ is fixed at 0.95. The proposed framework is implemented using the PyTorch platform. The experiments were conducted on a computer equipped with an Intel i9-13900HX CPU, an Nvidia GeForce RTX 4060 GPU, and 32 GB of RAM. To evaluate performance, we use classification accuracy (ACC), macro F1-score (F1), and area under the curve (AUC). Higher values in these metrics indicate better performance.

### 4.3 Experimental results and analysis

#### 4.3.1 Classification results and display.

In this experiment, to assess the classification performance of our method, this work compares our method with six competitive semi-supervised multi-view classification methods on four benchmark datasets. The proportion of labeled samples is set to 5%, 10%, and 15%. The experimental results ACC, F1, and AUC scores are recorded in Tables 1–3, respectively. For ease of observation, the best results are bolded in this work. Based on the experimental results, the following points can be observed:

(1) The results for ACC and F1 scores indicate that while traditional and deep methods each have their strengths on different datasets, the proposed method consistently delivers superior performance across nearly all datasets. For instance, on the Scene15 dataset, which has only 5% labeled samples, our method surpasses the second-best approach by 5.07% in ACC and 5.61% in F1. This outstanding performance is attributed to the dynamic fusion strategy in the high-level semantic space, which minimizes conflicts between reconstructed and category-specific features and reduces the adverse effects of low-quality views.
(2) From the results of AUC in Table 3, it is found that the method in this work achieves the optimal performance on all datasets, indicating that the proposed method in this work has good robustness and better discriminative ability, and the learned representations have a clear classification structure.
(3) SMILE-L demonstrates superior classification performance compared to the six comparison methods across almost all datasets, highlighting its ability to effectively extract task-related features while reducing redundant features. Furthermore, our method, SMILE, outperforms SMILE-L, underscoring the necessity and effectiveness of training the model with unlabeled data. This improvement stems from the inclusion of unlabeled data, which provides valuable information. Leveraging this information allows the model to gain a more comprehensive understanding of the data distribution, thereby enhancing the performance of downstream tasks.

**Table 1:** Accuracy results (%) compared among methods, the LACK algorithm cannot work on the GRAZ02 dataset and is replaced with “—”. The best results are highlighted in bold.

Ratio	Datasets	AMGL	MVAR	MLAN	JCD	LACK	IMvGCN	SMILE-L	SMILE
5%	handwritten	89.05(0.00)	95.53(0.00)	**97.52(0.14)**	95.95(0.00)	91.26(0.00)	95.76(0.15)	96.35(0.35)	96.41(0.66)
	Scene15	45.50(0.00)	57.34(0.00)	30.41(18.94)	58.78(0.00)	37.37(0.00)	52.49(2.06)	61.36(5.78)	**63.85(1.00)**
	Out_Scene	37.63(0.00)	44.06(0.00)	45.09(0.36)	68.80(0.00)	69.35(0.00)	72.89(0.84)	76.28(0.91)	**77.86(0.66)**
	GRAZ02	49.32(0.00)	53.68(0.00)	54.07(0.26)	55.32(0.00)	—	55.00(0.19)	56.49(0.41)	**57.04(1.50)**
10%	handwritten	92.39(0.00)	96.67(0.00)	**97.53(0.20)**	97.22(0.00)	91.83(0.00)	96.14(0.12)	97.49(0.18)	97.51(0.50)
	Scene15	50.48(0.00)	62.49(0.00)	54.34(0.43)	66.46(0.00)	41.94(0.00)	56.74(1.74)	70.20(0.83)	**70.98(0.66)**
	Out_Scene	41.54(0.00)	33.64(0.00)	46.42(0.23)	73.72(0.00)	70.17(0.00)	76.45(0.83)	83.80(0.25)	**84.51(0.47)**
	GRAZ02	53.65(0.00)	56.97(0.00)	54.18(0.30)	56.82(0.00)	—	55.02(0.25)	61.26(0.31)	**61.71(1.25)**
15%	handwritten	94.59(0.00)	96.71(0.00)	97.45(0.40)	97.47 (0.00)	91.71 (0.00)	96.23(0.06)	98.00(0.12)	**98.08(0.41)**
	Scene15	55.63(0.00)	63.56(0.00)	58.14(0.25)	69.69(0.00)	42.69(0.00)	56.98(2.36)	72.25(0.23)	**72.77(0.32)**
	Out_Scene	45.94(0.00)	68.13(0.00)	50.18(0.35)	76.11(0.00)	70.54(0.00)	77.39(0.66)	83.96(0.88)	**85.64(0.61)**
	GRAZ02	54.23(0.00)	54.94(0.00)	52.07(0.95)	58.45(0.00)	—	54.76(0.46)	62.18(0.73)	**63.34(0.93)**

**Table 2:** F1 results (%) compared among methods, the LACK algorithm cannot work on the GRAZ02 dataset and is replaced with “—”. The best results are highlighted in bold.

Ratio	Datasets	AMGL	MVAR	MLAN	JCD	LACK	IMvGCN	SMILE-L	SMILE
5%	handwritten	88.98(0.00)	95.54(0.00)	97.52(0.14)	96.01(0.00)	91.25(0.00)	95.78(0.15)	96.35(0.36)	96.40(0.64)
	Scene15	43.12(0.00)	54.00(0.00)	29.44(17.83)	56.81(0.00)	36.92(0.00)	49.44(2.17)	60.05(6.62)	**62.42(0.96)**
	Out_Scene	36.23(0.00)	44.68(0.00)	40.37(0.78)	69.35(0.00)	70.05(0.00)	73.09(0.80)	76.65(0.93)	**78.26(0.71)**
	GRAZ02	48.52(0.00)	53.55(0.00)	52.36(0.50)	52.60(0.00)	—	53.56(0.27)	54.32(0.46)	**55.66(1.55)**
10%	handwritten	92.36(0.00)	96.67(0.00)	**97.54(0.19)**	97.23(0.00)	91.83(0.00)	96.16(0.12)	97.48(0.18)	97.52(0.50)
	Scene15	48.00(0.00)	58.90(0.00)	51.34(0.60)	64.29(0.00)	41.02(0.00)	52.94(2.00)	69.30(0.78)	**69.42(0.73)**
	Out_Scene	39.66(0.00)	34.19(0.00)	41.90(1.07)	73.91(0.00)	70.89(0.00)	76.73(0.83)	84.00(0.24)	**84.66(0.48)**
	GRAZ02	52.97(0.00)	57.16(0.00)	52.60(0.17)	55.60(0.00)	—	54.01(0.30)	59.07(0.35)	**60.94(2.12)**
15%	handwritten	94.56(0.00)	96.71(0.00)	97.46(0.39)	97.48(0.00)	91.70(0.00)	96.24(0.06)	98.00(0.12)	**98.08(0.41)**
	Scene15	53.09(0.00)	60.64(0.00)	56.04(0.15)	67.76(0.00)	42.26(0.00)	53.12(2.77)	70.89(0.30)	**71.30(0.38)**
	Out_Scene	44.93(0.00)	68.45(0.00)	47.68(0.23)	76.19(0.00)	71.28(0.00)	77.56(0.66)	84.16(0.83)	**85.86(0.61)**
	GRAZ02	53.66(0.00)	54.77(0.00)	52.53(0.73)	58.33(0.00)	—	53.67(0.52)	62.06(0.78)	**62.93(1.16)**

**Table 3:** AUC results (%) compared among methods, the LACK algorithm cannot work on the GRAZ02 dataset and is replaced with “—”. The best results are highlighted in bold.

Ratio	Datasets	AMGL	MVAR	JCD	IMvGCN	SMILE-L	SMILE
5%	handwritten	98.61(0.00)	99.25(0.00)	99.69(0.00)	99.60(0.00)	99.55 (0.01)	99.85(0.01)	**99.87(0.06)**
	Scene15	86.17(0.00)	91.47(0.00)	80.16(14.28)	92.71(0.00)	90.91 (0.39)	92.64(0.62)	**93.57(0.26)**
	Out_Scene	67.72(0.00)	78.74(0.00)	72.90(0.42)	92.98(0.00)	92.30(0.25)	96.73(0.14)	**96.75(0.31)**
	GRAZ02	69.89(0.00)	75.24(0.00)	76.31(0.47)	72.10(0.00)	77.70 (0.16)	77.47(0.33)	**78.76(1.52)**
10%	handwritten	99.12(0.00)	99.30(0.00)	99.58(0.13)	99.93(0.00)	99.59(0.00)	99.90(0.00)	**99.92(0.04)**
	Scene15	88.92(0.00)	93.65(0.00)	91.69(0.24)	95.18(0.00)	92.07 (0.27)	94.64(0.09)	**95.64(0.40)**
	Out_Scene	71.30(0.00)	71.63(0.00)	74.53(0.06)	94.92(0.00)	94.70(0.58)	98.00(0.05)	**98.11(0.12)**
	GRAZ02	74.25(0.00)	77.05(0.00)	78.66(0.26)	81.41(0.00)	78.71(0.21)	82.00(0.28)	**82.54(0.37)**
15%	handwritten	99.32(0.00)	99.31(0.00)	99.57(0.20)	**99.94(0.00)**	99.58(0.01)	99.90(0.00)	**99.94(0.02)**
	Scene15	90.58(0.00)	94.37(0.00)	92.57(0.41)	96.03(0.00)	91.91(0.81)	95.27(0.05)	**96.11(0.18)**
	Out_Scene	72.37(0.00)	90.77(0.00)	75.04(0.36)	96.11(0.00)	95.02(0.41)	98.27(0.18)	**98.49(0.10)**
	GRAZ02	76.22(0.00)	75.78(0.00)	78.38(0.46)	81.80(0.00)	79.29(0.09)	82.30(0.15)	**83.47(0.58)**

#### 4.3.2 Visualization analysis.

**Fig 2:**
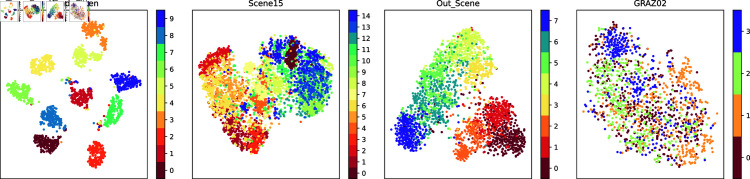
The t-SNE visualization of the original data.

**Fig 3:**
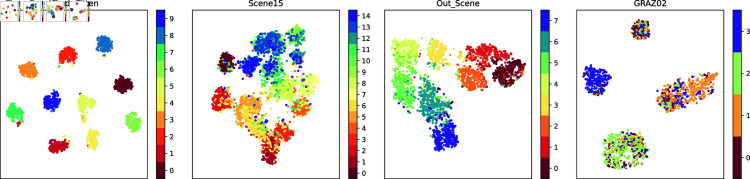
The t-SNE visualization of the fused features extracted in this work.

**Fig 4:**
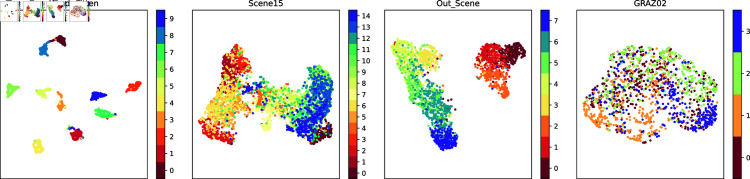
The UMAP visualization of the original data.

**Fig 5:**
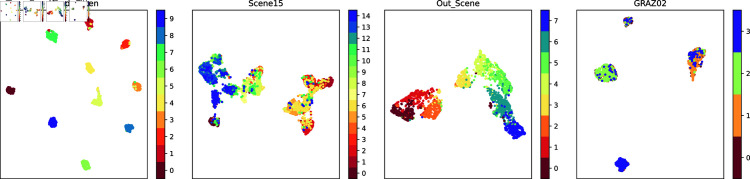
The UMAP visualization of the fused features extracted in this work.

In order to demonstrate the fusion representations learned in this work more intuitively, this work visualizes the original experimental dataset features and the fusion representations learned by this method using the t-SNE [49] and UMAP [49] dimensionality reduction methods. t-SNE focuses more on the local similarity, while UMAP focuses more on maintaining the global structure. The results of t-SNE visualization for the original view and the fused representation are shown in Fig 2 and Fig 4, respectively. The UMAP visualization results for the original view and the fused representations are shown in Figs 3 and 5, respectively.

Observation of the visualization results reveals that the raw data are distributed in a complex manner, with confusion between classes, making it difficult to clearly distinguish between different classes, especially on the two datasets Scene15 and GRAZ02. This suggests that the original data may have more overlapping and mixing in the high-dimensional space. The learned fusion representation, however, possesses obvious inter-class separation and intra-class compactness, indicating that our method is able to better distinguish different classes in the low-dimensional representation of the data, and has achieved significant improvement in data representation.

#### 4.3.3 Ablation study.

In this experiment, the work provides a further analysis of the importance of the high-level semantic mapping module and the dynamic fusion module used, in order to explore their key role in the model performance. The results of the ablation experiments are reported in Tables 4 and 5 to clearly demonstrate the impact of these two modules on the classification performance. The following conclusions can be drawn from the experimental results: Both modules play an active role in improving the classification performance of the model, indicating that the high-level semantic mapping module facilitates the extraction of classification features and avoids the influence of redundant features, while the dynamic fusion module avoids the influence of low-quality views and is able to deal well with the correlation and weight assignment between features of different views of different samples. The experimental results demonstrate the importance and effectiveness of these two modules.

**Table 4:** Ablation study of “w/” or “w/o” high-level semantic mapping module.

Dataset	w/	w/o
Handwritten	96.74	95.95
Scene15	70.18	67.84
Out_Scene	85.11	83.66
GRAZ02	62.7	61.16

**Table 5:** Ablation study of “w/” or “w/o” dynamic fusion module.

Dataset	w/	w/o
Handwritten	96.74	95.11
Scene15	70.18	68.58
Out_Scene	85.11	84.28
GRAZ02	62.7	60.56

## Discussion

The proposed method achieves dynamic fusion in multi-view settings under semi-supervised scenarios, which helps mitigate performance degradation caused by low-quality views. However, we acknowledge a key limitation of this work: the reliance on pseudo-label quality and the approximation accuracy of the true class probability (TCP). While pseudo-labeling is employed to address the scarcity of labeled data, the accuracy of these pseudo labels is inherently difficult to evaluate in the absence of ground truth. In extreme cases of low-quality views, the method may produce more inaccurate predictions, which could further compromise the TCP approximation and, in turn, hinder the extraction of view-specific features. Addressing the challenge of evaluating and improving pseudo-label quality remains an open and complex problem, and it represents a key direction for our future research efforts.

## Conclusion

With advancements in multimedia technology, the prevalence of multi-view data has increased, offering richer information for analysis and understanding while also presenting several challenges. Issues such as limited labeling information, effective multi-view fusion, and discriminative feature extraction need to be addressed. This work introduces a semi-supervised multi-view classification method based on dynamic fusion, which excels in extracting discriminative features and dynamically fusing multiple views from various samples. The high-level semantic mapping module reduces the impact of redundant features and retains important classification-related features, while the dynamic fusion module assigns weights to different views for each sample, minimizing the effects of noisy and low-quality views and exploring view associations effectively. Quantitative experiments validate the algorithm’s effectiveness and superiority, and visualization experiments demonstrate that the learned fusion features have a strong classification structure. Ablation studies highlight the importance and effectiveness of each module. Future work will focus on improving semi-supervised multi-view classification, enhancing pseudo-labeling accuracy, discovering more discriminative features, and achieving better fusion of multiple views for optimal classification representations.
